# A Tool to Simulate the Transmission, Reception, and Execution of Interactive TV Applications

**DOI:** 10.1155/2017/1834907

**Published:** 2017-01-18

**Authors:** Manoel Carvalho Marques Neto, Raoni Kulesza, Thiago Rodrigues, Felipe A. L. Machado, Celso A. S. Santos

**Affiliations:** ^1^Computer Science Department, Instituto Federal da Bahia (IFBA), Emidio dos Santos St., S/N, Barbalho, 40301-015 Salvador, BA, Brazil; ^2^Informatic Center, R. dos Escoteiros, Universidade Federal da Paraíba (UFPB), s/n, Mangabeira, 58055-000 João Pessoa, PB, Brazil; ^3^Computer Science Department, Universidade Federal do Espírito Santo (UFES), Fernando Ferrari Av. 514, Goiabeiras, 29075-910 Vitória, ES, Brazil

## Abstract

The emergence of Interactive Digital Television (iDTV) opened a set of technological possibilities that go beyond those offered by conventional TV. Among these opportunities we can highlight interactive contents that run together with linear TV program (television service where the viewer has to watch a scheduled TV program at the particular time it is offered and on the particular channel it is presented on). However, developing interactive contents for this new platform is not as straightforward as, for example, developing Internet applications. One of the options to make this development process easier and safer is to use an iDTV simulator. However, after having investigated some of the existing iDTV simulation environments, we have found a limitation: these simulators mainly present solutions focused on the TV receiver, whose interactive content must be loaded in advance by the programmer to a local repository (e.g., Hard Drive, USB). Therefore, in this paper, we propose a tool, named BiS (Broadcast iDTV content Simulator), which makes possible a broader solution for the simulation of interactive contents. It allows simulating the transmission of interactive content along with the linear TV program (simulating the transmission of content over the air and in broadcast to the receivers). To enable this, we defined a generic and easy-to-customize communication protocol that was implemented in the tool. The proposed environment differs from others because it allows simulating reception of both linear content and interactive content while running Java applications to allow such a content presentation.

## 1. Introduction

Digital technologies dissemination in many knowledge areas led to changes in implementation of most modern society activities such as work, education, health, and entertainment. A vehicle that has potential to play an important role in the use of these technologies is Digital TV. Among the main benefits offered by Digital TV, we can highlight audio and video quality improvement and the inclusion of new services such as interactive TV (iDTV). The main feature of these systems is the capability of running applications that have been downloaded as part of the broadcast stream and synchronized with linear TV program: this is indeed what makes the distinction between an interactive TV system and other TV systems (basic digital or Smart TV) [1–3].

One of the key points for developing iDTV software is the existence of an environment to simulate the transmission, reception, and execution of such software prior to their deployment in a real environment. This paper relies on the definition of such environments as simulators: “a device that enables the operator to reproduce or represent, under test conditions, a phenomenon or process in the same way as (or as closely as possible) it occurs in the real world” [4, 5]. The major reasons for the use of simulation environments are cost reduction to purchase real environments, increasing the speed of development, and security (once an application is under test could only cause harm to a virtual environment).

Commonly, the simulators available for developing iDTV software allow running such artifacts on computers to simulate only the TV receiver side [7–9]. They do not allow simulating a TV station, responsible for broadcast audio and video content and also the iDTV software and data, sometimes named Extra Content. This limitation is an obstacle to simulate “interactive TV applications” (iTVA) whose simulation has as main requirement the dynamics for transmission, reception, and reproduction of content.

To fill this gap, the main objective of this paper is to present a tool, named BiS (Broadcast iDTV content Simulator), that allows the transmission, reception, and execution of iTVA. The transmission and reception of iTVA are performed through a TCP/IP network using a broadcast method based on data carousel (in the same way as real in the world). In BiS tool, the receiver module is also responsible for running an iTVA implemented in JavaDTV [10]. Importantly, this tool does not include the content production phase and also does not consider performance issues in content transmission (time delay, bandwidth, content quality, etc.). These issues are difficult to assess since the communication network in a real TV environment has very different aspects of a TCP/IP network used by the simulator. For example, the TCP/IP network uses a shared link while the TV network uses a dedicated link. Therefore, the focus of the BiS tool is only to simulate the environment dynamics for transmission, reception, and execution of interactive TV applications.

To validate and demonstrate the main features of the tool, we have developed, transmitted, received, and ran three Java applications (named Xlets [10]). They present during the transmission of a linear TV program (i) replays sent by a broadcaster, (ii) weather forecast obtained both from a broadcaster and from an Internet Service Provider (ISP), and (iii) photos of fighters during a Mixed Martial Arts (MMA) fight transmission. The simulated applications can be viewed on YouTube (http://youtu.be/lJ6ZPxgf-CI) (http://youtu.be/VRg6149XesU). Moreover, the source code of the BiS tool is available for download on the web (https://github.com/manoelnetom/IDTVSimulator).

The next section of this article presents some concepts and definitions of iDTV and the related work to this paper. The third section presents the BiS tool and the main functionalities. The fourth section presents its architecture. In the fifth section, the experiments used to validate and demonstrate the main features of the simulation environment are presented. The sixth section presents the conclusions and the possible evolution of this work.

## 2. iDTV Basic Definitions

Some iDTV previous definitions are important to improve the comprehension of this paper. This section aims to fulfill this role.

In computer science world, “program” is synonymous with “software.” However, in TV world, the most appropriate meaning for this word is “linear program.” This paper relies on the definition provided by [2], where such programs are defined as multimedia applications through which a viewer can interact via remote control. It means that, in addition to audio/video main streams (linear program), the viewers can receive (from broadcaster) or download (from web) software that allow then interacting with the delivered and displayed content on a TV screen or on a second device screen [11]. These software are defined as “applications” designed to execute on a TV receiver and to manipulate/consume some data (e.g., video, audio, text, and XML). Such applications and the data manipulated/consumed by them are defined as “interactive content.”

**Figure 1:**
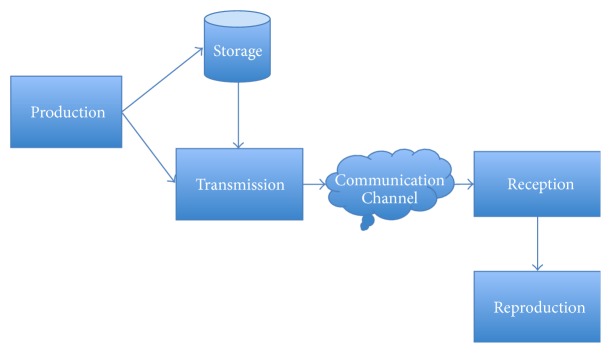
Basic phases involved in the creation, transmission, and presentation of interactive content [6].

Commonly, the basic activities related to interactive content are grouped into 4 phases [6]:* production*,* transmission*,* reception*, and* reproduction* (see Figure 1).

The* production* phase may include (i) media creation activities (e.g., creating images with different available configuration settings, use of television screen area, and sound quality adjustment), (ii) application development activities, and (iii) the integration between a linear TV program and an application.

Some remarks should be highlighted on the production phase. First of all, the media creation activities generate a set of artifacts that can be* stored* and reused many times in a recorded linear TV program or can be generated and used in a live linear TV program. Furthermore, the application development activities consist in building a set of synchronized compositions of nodes. These compositions may represent media (such as video, audio, text, and images), data, and programming language objects to be presented/executed during a linear TV program. These synchronized compositions may be specified using a programming language such as Lua [12], JavaTV [13], JavaDTV [10], Broadcast Markup Language (BML) [14], and Nested Context Language (NCL) [15]. In this context, the use of authoring tools is useful to abstract the complexity of such languages.

The* transmission* and* reception* phases are executed through MPEG-2 transport stream and data carousel (defined by DSM-CC: Digital Storage Media-Command and Control) [16]. The DSM-CC is an ISO/IEC standard [17] (included on MPEG-2) used by digital terrestrial TV systems (the focus of this work) and developed for the delivery of multimedia broadband services.

The DSM-CC is an important mechanism for interactive content generated in a broadcaster. It is responsible for sending the interactive content in a cyclic way (data carousel). This content is multiplexed together with a linear TV program in a MPEG-2 transport stream. It also works as a virtual disk that stores interactive content to be delivered to viewers. Through the DSM-CC we can set on the receiver the same directory structure used in the content generator. With the cyclic sending, the interactive content can be correctly transmitted to the receiver even if the channel is tuned long after the beginning of a linear TV program.

Another way to transmit interactive content data to a TV receiver is through the return channel. This mechanism should be used for two-way communication with the broadcaster and thus potentially with other content providers (e.g., ISP). Interactive TV standards theoretically support loading applications from return channel. We say “theoretically” because they do rely on a broadband return channel and advanced receiver implementation, and so this is not likely to be widely adopted in practice. However, many broadcasters send some additional data for the interactive application through the return channel. Sync events with media content, files (e.g., the main class manifest) and events related to signaling and application security are usually sent by the broadcast channel. In this paper we use this approach.

If an interactive content with a specific TV program, it is frequently helpful to be able to synchronize the behaviour of application to the events on scenes. In numerous multimedia authoring systems, this is implemented based on the concept of a timeline where these changes in execution take place at a specific time. The problem with interactive TV applications is that there is no real concept of media time when dealing with a broadcast MPEG-2 stream. As we can join the stream at any point, we have no method of knowing how long it is since the stream started (or even what the idea of “starting” means in a broadcast sense). This means that we cannot utilize media time as a method for synchronizing applications to their related media.

Although DSM-CC is often used as a broadcast file system protocol, there is more than that. We use some extra capabilities outlined by DSM-CC to support the synchronization, especially for stream events. These are markers that are inserted in a transport stream by way of MPEG-2 private sections, with each event consisting of an identifier and a time reference. Stream events will be detailed in Section 4.1.1.

The* reproduction* phase includes the presentation of interactive content with linear content. This task means running a multimedia presentation (with time and space synchronization constraints) or run software written in a procedural language (which also implements synchronization constraints). Usually, in terrestrial Digital TV Systems, this is implemented on the middleware installed in the DTV receivers or set-top-boxes. Examples of middleware for Digital TV include Ginga [18], Multimedia Home Platform (MHP) [19, 20], and DASE-ATSC [21].

Another important definition to this paper is how to classify interactive content. According to [22], the interactive content may be divided into three groups.

The first group includes the interactive content that has no relation with the linear content presented on TV (e.g., E-mail or TV-banking presented during a film). In this group, the interactive content could be sent by the broadcaster (using the DSM-CC) or downloaded from any other content provider (through the interactive channel). The only difference between iDTV applications (included in this interactive content) and, for example, PC applications is to how to fulfill nonfunctional requisites such as “execution platform” (TV) and “input and output mechanisms” (remote control). To simulate this environment, we can use a player that present the interactive content loaded from a local repository (e.g., USB stick). Therefore, the transmission and reception aspects are not fundamental for this group.

The second group includes the interactive content that have a relation with a recorded linear TV program (whose content is known in advance) and with strong time synchronization constraints, for example, interactive advertising of products presented in specific moments of a movie. In this group, the interactive content related to the movie is usually sent by the broadcaster using the DSM-CC. However, as in the first group, we can also simulate this environment by using a player that present the interactive content loaded from a local repository and not considering the transmitting/receiving aspects, since both the linear TV program (or content) and the data (including synchronization constraints) are known in advance.

The third group includes a live generated interactive content that has a relation with a live linear TV program content and with time synchronization constraints that need to be accomplished during the presentation. In this group, the interactive content is also sent by the broadcaster using the DSM-CC. Due to the synchronization constraints built at runtime, we must consider aspects that directly influence the process of transmitting, receiving, and running such interactive content to simulate this environment.

This paper relies on the classification of such interactive content (2nd and 3rd groups) as interactive TV Applications (iTVA). The main contribution of this paper is to fill the gap by creating a “complete” simulation environment that allows transmitting, receiving, and presenting/running the interactive content included in the previously mentioned groups.

### 2.1. Simulation Environments Related to iDTV

The Set-Top-Box Virtual Ginga-NCL is a virtual machine, built over VMware [23], aimed to facilitate the distribution and deployment of the Ginga-NCL player. This player version was written in C ++ and has the most advanced presentation features for declarative interactive content, better performance, and implementation similar to those that are embedded in real set-top-boxes [7, 24].

To present an interactive content on Virtual Set-Top-Box, the option is to use a remote file transfer application like SFTP (SSH file transfer protocol) [25]. As a matter of fact, all control commands that are supported by this environment are performed through SSH remote console application. The installation of interactive content, for example, is made using this interface. The treatment of interactive events triggered by users is done through the PC keyboard. The keyboard keys are mapped to specific functions of an interactive content simulating the buttons of a remote control.

The simulator is able to run any interactive content written in NCL and faithfully reproduce a real execution environment for such content. It allows the presentation of contents but does not explore the aspects related to its transmission. Therefore, the interactive content should be available before presentation in this simulation environment.

The XleTView is considered one of the most popular tools to simulate Digital TV on a PC platform. It includes the execution of Java applications (named Xlets) and implements the MHP APIs [19] and the API JavaTV [13]. To simulate the presentation of the linear TV program, the XleTView allows a user to select a video file whose content is displayed in a rectangular and central part of the simulator window. Likewise, the user can select interactive content written in JavaTV that will run over an invisible panel located on the Main Content screen. The simulator provides a virtual remote control that allows handling interactive events located on the right side of the window. The XleTView is focused only on running interactive content. In this tool a major limitation is the inability to simulate the transmission/reception of content sent by a broadcaster. It does not implement the DSM-CC or any other similar mechanism.

The OpenMHP is a simulator whose purpose is to run Xlets on a PC (very similar to the XleTView). It is also based on MHP specification and uses JavaTV and JMF (Java Media Framework) APIs implementation [8, 26]. The OpenMHP offers a textual output with debug information that is responsible for presenting all events and messages for the simulated applications at runtime. This debugging functionality offers a better view of test applications developed. Thereby, when compared to XleTView, the OpenMHP can be considered a more complete simulation tool. However, the lack of functions to transmit/receive interactive content [17, 27–29] and the complexity of its installation and configuration are the two major limitations of OpenMHP.

The Ginga-J Emulator is an open source project developed in Java whose architecture is also based on XleTView [9]. One of the main innovations of Ginga-J Emulator was conducting the reference implementation in accordance with the new API JavaDTV [10] adopted by the middleware Ginga. Despite being based on a particular platform, this tool not only works as a proof of concept, as it also allowed identifying limitations of the existing XleTView software architectures. Some of these difficulties must be considered in order to simplify the process of porting and extend the platform.

This emulator provides an execution environment easy to install and use on a PC. However, the tool has limitations to represent the features available in a real set-top-box. The main restriction is related to the use of network protocols such as signaling applications, broadcast file system, media and interactive content synchronization, selection of elementary streams, and access to service information and data carousel, since the application execution is performed locally. In other words, the Ginga-J Emulator does not allow simulating neither the transmission nor the reception of interactive TV applications.

The FrameIDTV [30] is a framework used to implement interactive content (or portion thereof). It was included in the list of related works of this paper because it provides a set of objects that allow building interactive content (in Java) that use communication over a computer network. To support this feature, this framework specifies a generic and easy-to-customize application layer protocol. Thus, if a developer wants to use the FrameIDTV communication services, he should include in an interactive content the FrameIDTV objects that are responsible for such communication.

### 2.2. Remarks and Discussions

As is shown in Table 1, none of the tools described in this section cover all the basic activities related to interactive content: production, transmission, reception, and reproduction. None of them covers the production phase. Most of them cover the reproduction of interactive content on computers to simulate the TV receiver. Only one of presented tools (FrameIDTV) covers aspects of transmission/reception but still without following the DSM-CC dynamics. Finally, none of the work allows simulating the interactive content presentation included in the third group.

One of the reasons for the lack of simulators that include the production phase may be the chain of available tools. Currently, the tool chain for the production phase is quite mature. Some examples of these tools include NEXT [31], Composer [32], T-MAESTRO [33], iTV Project [34], AthenaTV [35], and Cacuria [36]. Thus, we decided in the same way to not include the production phase in the proposed simulator. However, including this phase is one of the possible future works of this project.

The fact that most simulators presented here allow the reproduction of interactive content, combined with the desire to produce a tool aligned with the standard adopted in Brazil (ISDB-Tb: Integrated Services Digital Broadcasting, Terrestrial, Brazilian version) [6, 37], led us to reuse and extend the Ginga-J Emulator to meet the reproduction phase. The Ginga-J Emulator does not address the synchronization aspects between linear and interactive content (see Section 2.1) conformance with ISDB-Tb. Its limitation is exactly the aspects of transmission and reception.

Another important discussion for this work is to highlight some aspects of FrameIDTV. First, the FrameIDTV does not provide a reproduction environment. It focuses on design and implementation details of interactive content. Another aspect is the communication protocol defined in the FrameIDTV components. These components follow the client-server paradigm with transport over TCP. This approach assumes that there is always a bidirectional communication flow between a client and a server. The communication model used by FrameIDTV (push/pull) is the same used on the Internet. In fact, this aspect is a limitation of FrameIDTV framework. In a conventional TV environment (without interactive channel), the transmission of the content is always done by the TV station in broadcast mode. The TV station uses the pull communication model [38], where content is transmitted simultaneously to all receivers tuned in a specific channel.

## 3. BiS: Broadcast iDTV Content Simulator

This section presents the implementation details of the tool named BiS: Broadcast iDTV content Simulator. The focus of the BiS tool is the transmission, reception, and reproduction phases. It was created from the source code of the Ginga-J Emulator and this allowed reusing the functions to present interactive content written in JavaDTV. It allows presenting interactive content from any of the three groups defined in Section 2.

The major differential of the BiS tool is exactly the main limitation of most of the related works: the use of broadcast protocols (applications signaling) and DSM-CC (broadcast file system and media and interactive content synchronization). Therefore, the BiS tool includes functions for transmission and reception of contents and uses a broadcast method through a data carousel to simulate the transmission of both linear TV content and interactive content. In addition, the BiS tool allows users to tune a channel and consume contents sent by this channel or other content providers.

Importantly, despite being a simple solution, it has never been implemented and made available to a simulator. The importance of simulating the environment which enables the transmission, reception, and execution of interactive TV applications are (i) evaluating the synchronism between linear TV content and interactive content sent to the receivers and (ii) the possibility of simulating the sending of content generated live during the presentation of a linear TV program. In other words, it is not necessary to know the entire linear TV program content in advance and, still, you can simulate the live broadcast of interactive content related to the linear TV program being presented.

During the design phase of this project, the list of functional requirements was mapped in the following use cases. They have two main actors: the Broadcaster (Director, Editor, etc.) and the Viewer and will be presented in the following two groups:

1. Actor:* Broadcaster*
  a. *Configure Network Information*. Before any action, an Actor must provide the settings necessary to send contents. Through a configuration dialog which should be displayed when starting the simulator, the Actor should be able to enter the following data:
    - broadcast IP;
    - network port to send linear TV content;
    - network port to send interactive content;
    - channel name.
  The other features of the simulator should only be released after defining these settings.
  b. *Transmit Linear TV Content*. An Actor must select a video file to be transmitted. This video will be used as linear TV content. For this functionality, the simulator must use the VLCj API that must be configured to transmit over UDP on network ports and IPs previously defined.
  c. * Start Data Carousel*. An Actor must be able to initiate the data carousel at any time even though he has not added any content yet. This option must be disabled when the carousel is running.
  d. * Stop Data Carousel*. When stopping the data carousel, all contents added to it must be removed. This feature represents the inclusion of the use case below:
    - * Restart Data Carousel*. An Actor must be able to reset the carousel at any time. To reset the carousel, a user has to stop it, so that all contents will be removed, and then the BiS tool automatically restarts it again.
  e. * Pause Data Carousel*. Once the carousel is started, an Actor can pause it at any time. In this case, the contents are not removed, and when the carousel is restarted, the transmission must go back to the point where it stopped.
  f. * Update Contents List.* The functions for insertion, removal, and replacement of contents must automatically update the simulator window that displays the contents. This feature represents the inclusion of the three use cases below:
    i. * Add Contents to Data Carousel*. An Actor must be able to add contents to the carousel, at any time and even if the carousel has not been started, displaying these contents in a list to the Actor with the following data:
      A. content name;
      B. version;
      C. content type (Automatic, Notify or List).
    This function can be used even with the object carousel started.
    ii. * Remove Contents from Data Carousel*. An Actor must be able to remove contents from the carousel at any time. This action must update the graphic component which lists all the contents in carousel.
    iii. * Replace Contents from Data Carousel.* An Actor will be able to replace, at any moment, contents previously added to the carousel. This action must update the version information of these contents in the window where they are displayed.
2. Actor:* Viewer*
  - * Tune a TV Channel*. This use case represents the simulation of “to tune a channel in a conventional television.” In the BiS tool, this feature is mapped to the action of choosing a pair of network ports for receiving data through the network. In this context, one port is used for receiving linear content and the other for receiving interactive content. This use case includes the following.
    - * Receive Linear Content*. This use case allows simulating the reception of linear TV content. An Actor has no control over this content. As in a conventional broadcast television, a viewer can only tune a channel and watch the content. In this context, he cannot forward, pause, or rewind a video stream.
    - * Receive Interactive Content*. This use case allows simulating the reception of interactive content. This content is classified into three distinct types that must undergo special treatment:
      - * File*. Digital files with any type of content should be stored in the receiver's Hard Drive.
      - * Directory*. For directories, the process comprises receiving the entire tree of files (directory, subdirectories, and files) and stores it on disk respecting the original hierarchy sent.
      - * Application*. For applications, the process comprises receiving and storing the entire file structure and metadata of the application. These metadata includes (a) application name and (b) application startup class. These metadata are also kept in memory for later execution.
  - * Run JavaDTV Applications*. This use case includes the Receive Interactive Content use case. It defines the fact that if the interactive content is an application, it must be executed.

## 4. Architecture

**Figure 2:**
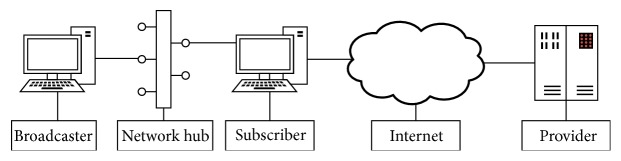
Architecture of the simulator.

Figure 2 shows the simulator architecture. It divides the simulation into three major modules: (1) Broadcaster, which is the transmitter side of the simulation environment, (2) Subscriber, which represents the receiver side, and (3) Provider, which represents other content sources than broadcaster, for example, a video portal on the Internet, and news feed. The following topics detail each of these modules.

The Broadcaster module is associated with the* Transmission* phase (see Section 2) and it covers functions defined in the first group of use cases in Section 3. It was divided into two other modules: (i) Main Content provider and (ii) Extra Content provider.

Likewise, the Subscriber module is associated with the* reception*/*reproduction* phases (see Section 2) and it covers functions defined in the second group of use cases in Section 3. It was divided into three modules: (i) Extra Content Subscriber, (ii) Main Content Subscriber, and (iii) Presentation. The following topics detail each of these modules.

### 4.1. Broadcaster

**Figure 3:**
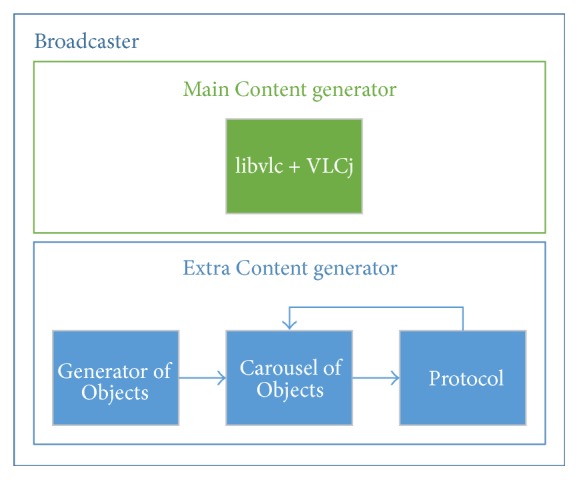
Broadcaster architecture.

Figure 3 shows the Broadcaster architecture. This architecture can be represented by two modules called “Main Content generator” and “Extra Content generator.”

The Main Content generator module is responsible for transmitting audio and video which may represent the linear TV content. Thus, this module uses the library* libvlc*, included in Video LAN media player (VLC) project. This library was used to implement the functions that allow transmission, reception, and visualization of such content. The VLC project also offers the VLCj API which allows using code written in Java to handle transmission, reception, and media encoding functionalities [39]. The module uses an exclusive network stream (through a UDP service and using broadcast method) in order to simulate a real environment of Digital TV. Once loaded, the module is able to transmit linear TV content, using a video file selected by a user.

The Extra Content generator module is responsible for transmitting interactive content that includes applications and data (e.g., text, XML, image, audio, and video consumed by such applications). The interactive content is transmitted cyclically, in another network stream (other than linear content), but still uses broadcast method. The transmission dynamics of interactive content was inspired on DSM-CC used by terrestrial Digital TV systems. The carousel implemented in this work aims to control the data flow delivery for different types of interactive content. For this purpose, first, each content is mapped into a carousel object and, then, each object is stored in a circular queue. After that, the carousel transmits pieces of each object (packets) using a time interval size proportional to the number of objects in the queue. The Extra Content generator module was subdivided into three submodules: Generator of Objects, Carousel of Objects, and Protocol. These submodules will be detailed below.

#### 4.1.1. Generator of Objects

This submodule is responsible for identifying the type of content transmitted and also trigger events that can be used to synchronize the linear TV program and interactive content. For this, it implements functions that allow recursively browsing directories with contents, allow classifying files found on such directories, allow mapping them into carousel objects, and allow stream events to be inserted into linear TV program easily.

**Figure 4:**
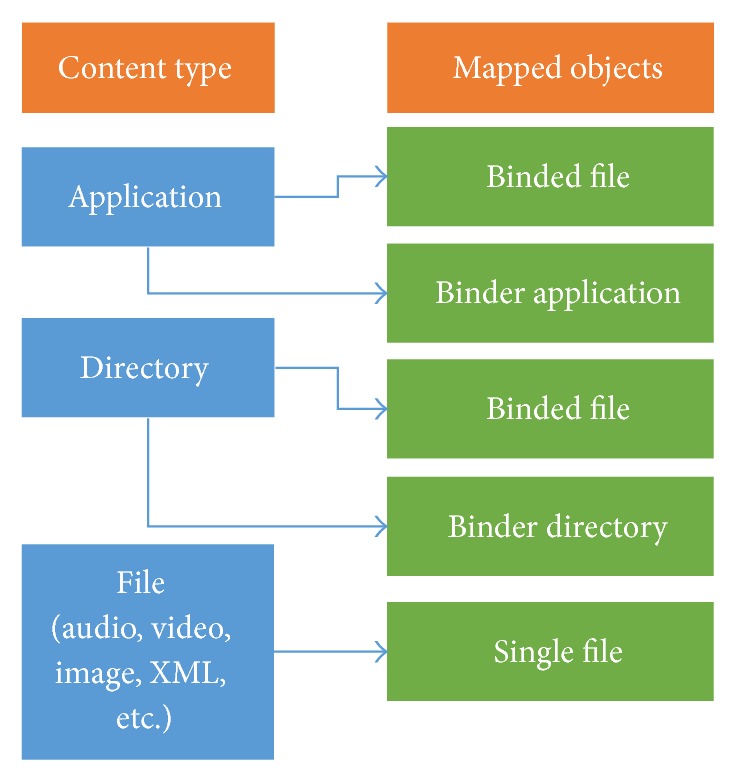
The list of content types and their respective mappings on the carousel of objects.

Figure 4 shows the list of content types and their respective mappings on the carousel of objects. A selected content should be identified as “Application,” “File,” or “Directory.” If a selected content is a Java file, this module will identify it as an* Application*, if the content is a folder, it will be identified as a* Directory* and if the content is anything different from a file or a folder, it will be identified as a* File*.

The contents identified as* Directory* can be mapped into objects “Binded File” and “Binder Directory.” The contents identified as* Application* can be mapped into objects “Binded File” and “Binder Application.” Simple file (identified as* File*) can be mapped only as “Single File” objects. The objects defined in this work are similar to those defined on the DSM-CC real implementation.

A “Single File” object is equivalent to a* File* object on DSM-CC real implementation. It represents a data file without any connection with other files to be transmitted. The “Binded File” object represents a file that is semantically linked to other files. This indicates that access to this file should only occur when all linked files have been received. A “Binder Directory” object has the same functionality as the* Directory* object on DSM-CC. It represents a directory that should be created in a receiver and saves the list of files that are stored in such a directory. The “Binder Application” and “Binder Directory” are similar to each other. It (“Binder Application”) represents a directory that contains an application and was created only to facilitate identification of the transmitted applications. Besides containing all the data of a “Binder Directory” object, the “Binder Application” contains the application name, starter class name (main class), and the type of execution. The types of execution supported by the BiS tool include the following.

1. *Automatic*. An application should start automatically as soon as it is received by a receiver.
2. *Notify*. An application should not be started automatically at a receiver. However, a user should be notified about each new content. To access it, a user must start the application from a list of applications on a receiver.
3. *List*. An application should only be added to the list of applications on receiver.

Stream events, as we saw in previous section, are markers consisting of an identifier and a time reference. The identifier allows each stream event to be unambiguously identified, while the time reference indicates at what point in the stream the event should trigger. The transport stream might contain a data carousel which includes a set of stream event objects. These identify each event by a textual name and allow the mapping of this name to the numeric identifier contained in the event itself. This lets an application programmer to know what events can get generated previously and helps it to verify that an application is registering itself as a listener for broadcaster events. The submodule is responsible for managing CRUD operations (add, remove, edit, and query stream events by id or name) of stream events that will be sent in the broadcast stream.

#### 4.1.2. Carousel of Objects

After mapping objects (in the previous submodule), the Carousel of Objects module receives the set of objects (that represents the contents) and stream events and adds it to the carousel and transport stream on the simulator, respectively. Each object and stream events that will be broadcasted will be added individually and each will have its own time frame to be transmitted.

The implementation of carousel performed in this work was based on Java libraries for data transmission through network (e.g.,* Socket*,* DatagramSocket*). In order to maintain similarity with DSM-CC and enable reliable delivery (not using TCP), a protocol that enables integrity checking and object type checking was defined and implemented. This protocol will be detailed below.

#### 4.1.3. Protocol

This section describes the protocol defined and implemented in this work. The Protocol submodule defines the format of messages exchanged between the Broadcaster and the Subscriber modules. It contains the representation structures (e.g., protocol headers) and the conversion functions required to execute the transmission (and also reception) of contents. The Protocol submodule is located at Broadcaster module, but it is important to note that the Subscriber module also uses the services offered by this submodule. Initially, it uses functions that receive the selected objects as parameters and transform them into an array of* bytes*. After that, the “Carousel of Objects” submodule performs the data transmission through network. The receiver (Subscriber module) must perform the reverse process, transforming the* bytes* received in objects again.

The first message format defined by this protocol is the data packets header used on the streaming of contents. This header is detailed in Table 2.

1. “ID” is an 8-byte size field that is used to provide a unique identifier for each object added to the carousel. For example, if an application is composed by a file whose extension is “.class” and two more XML files, all three files are mapped as carousel objects and receive unique identifiers. Thus the receiver can treat each element individually and check their consistency.
2. “Type” is a 2-byte-size field that is used to identify contents and to map them as carousel objects. As described in Section 4.1.1, there are five possible values for the “Type” field: “Binded File,” “Binder Directory,” “Binder Application,” “Single File,” and “Stream Events.” The latter two represent stream event objects.
3. “Version” is a 2-byte-size field that is used to define the version number of an object. In some cases a substitution of a transmitted content may be required. In this context, new objects should replace old ones keeping the same ID (only updating their version numbers).
4. “Number of Packets” is an 8-byte-size field that stores information about the total number of packets that compose an object. This information is used to determine whether an object has been completely transmitted.
5. “Packet Sequence” is an 8-byte-size field that stores a number used to identify and sort each of the packets.
6. “Data Length” is a 4-byte-size field that is used to store the content size indeed loaded in each packet.
7. The last packet field is named “Data.” It is a 30720-byte-size field that is used to store the content loaded by a packet.

Particularly for “Binder Application” objects, “Data” field defines a structure to facilitate the execution of applications. This data structure is shown in Table 3. It shows the information that is transmitted in the Data field for “Binder Application” objects. The first* byte* indicates the type of execution. The second* byte* is related to the amount of* bytes* used to the name of the main class. This name can use up to 250* bytes* (3rd at 252). The 253* bytes* represents the amount of* bytes* used to the application name. To this, it is possible to use up to 32* bytes* (from 254 to 285). After that, there are 4* bytes* to represent the amount of identifiers. The area reserved for the transmission of identifiers have size equal to 30430 bytes (from 290 to 30720) which can represent up to approximately 3803 identifiers (“Binded File” objects).

From a protocol implementation perspective, stream events are split into two parts: “stream event” and “stream event descriptors.” “Stream events” are stored in an object carousel and are just like any other objects. The “stream event descriptors” are inserted in the broadcast stream as markers and tell the receiver that an event has actually been embedded. A “stream event descriptor” contains three main attributes: the ID of the object, an NPT (Normal Play Time) value at which the event should be triggered, and several application data. The ID allows resolving which stream event object is related to this descriptor in receiver side. Since a transmitter cannot be sure just where a stream event descriptor is added to a stream, each descriptor transports an NPT value that says when the event should be notified. This enables the application programmer to know in advance that it should generate an event when a specific NPT value is touched, giving a further predictability. The iDTV standards specifications state that every stream event must be signaled at least once every second for a minimum of five seconds earlier than the time they should trigger. In this case, these repeated objects must have the same values.

In addition, a “stream event descriptor” can also be configured to start immediately—these are called “do it now” events. This allows stream events to be inserted into a live content much more easily. In this case, events are only broadcast once per time, and so some precautions should be taken by the application programmer to ensure that it receives them rightly. This “Carousel of Objects” submodule configures the stream event descriptors of the registered streams events objects in “Generator of Objects” submodule.

### 4.2. Subscriber

**Figure 5:**
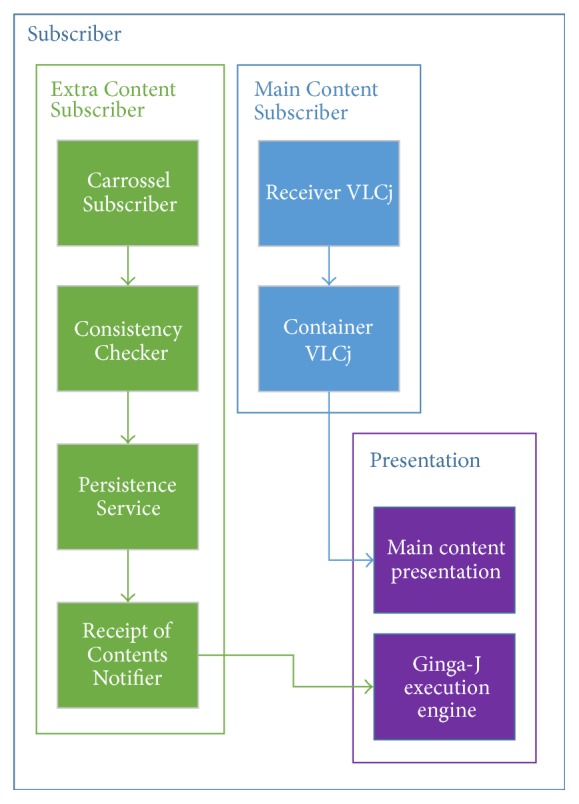
Subscriber architecture.

The Subscriber architecture is divided into three modules called Extra Content Subscriber, Main Content Subscriber, and Presentation as illustrated in Figure 5. The following topics detail each of these modules.

#### 4.2.1. Extra Content Subscriber

This submodule aims to receive interactive content, to verify its integrity, to persist it on receivers, and to notify other submodules about the availability of a new content and stream events. For this reason, such a module is divided into four parts: Carrousel Subscriber, Consistency Checker, Persistence Service, and Receipt of Contents Notifier.

The Carrousel Subscriber aims to allow the reception of data obtained from a Broadcaster. For this, it uses Java libraries for data transmission over network (e.g., Socket, DatagramSocket) and processes packets (messages) whose format was specified in the protocol defined in this work (see Section 4.1.3). Each packet received by this carousel is sent to the Consistency Checker.

The Consistency Checker is an integrity check protocol that aims to maintain similarity with the DSM-CC and to ensure reliable delivery of packets (without using TCP). This means that it checks the integrity of packets received from Carrousel Subscriber avoiding the receipt of duplicate packets and, as a consequence, the consumption of incomplete objects.

**Figure 6:**
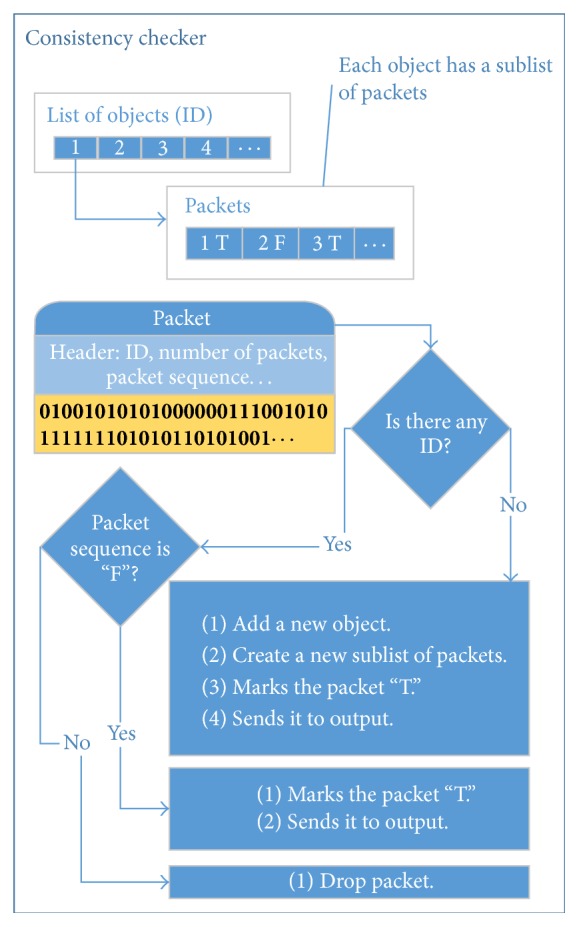
Consistency Checker.

The entire process executed by the Consistency Checker submodule can be viewed in Figure 6. To check the consistency of received packets, the Consistency Checker has a list of identifiers that represents each object whose receiving process has already started. Each object of this list contains a sublist with a tuple: packet identifier and a Boolean variable that identifies whether the packet was received (true) or not (false). Thus, the process to check the consistency implies in browsing the list of objects and, for each of these objects, performing the reading of the received packet header. In this phase only the following fields are evaluated: ID, number of packets, and packet sequence. From this point, three situations may occur:

1. If the list contains no object whose identifier is equal to the received packet header's ID field:
  - The Consistency Checker adds a representation of the new object to the object list.
  - The Consistency Checker adds the newly created object to the list of packets that goes from 1 to the value of Number of packets contained in the received packet header field. All positions on the list are marked as packet not received (false).
  - The Consistency Checker marks the newly created packet, whose identifier is equal to the received packet Sequence field, as “received” (true).
  - The Consistency Checker sends the packet to the output.
2. If the object list contains an object whose identifier is equal to the received packet header's ID field:
  - The Consistency Checker marks it as “received” (true).
  - The Consistency Checker sends the packet to the output.
3. If the object list contains an object whose identifier is equal to the received packet header's ID field and the representation of the packet (in the packet list) is marked as received (true), then this packet is dropped.

**Figure 7:**
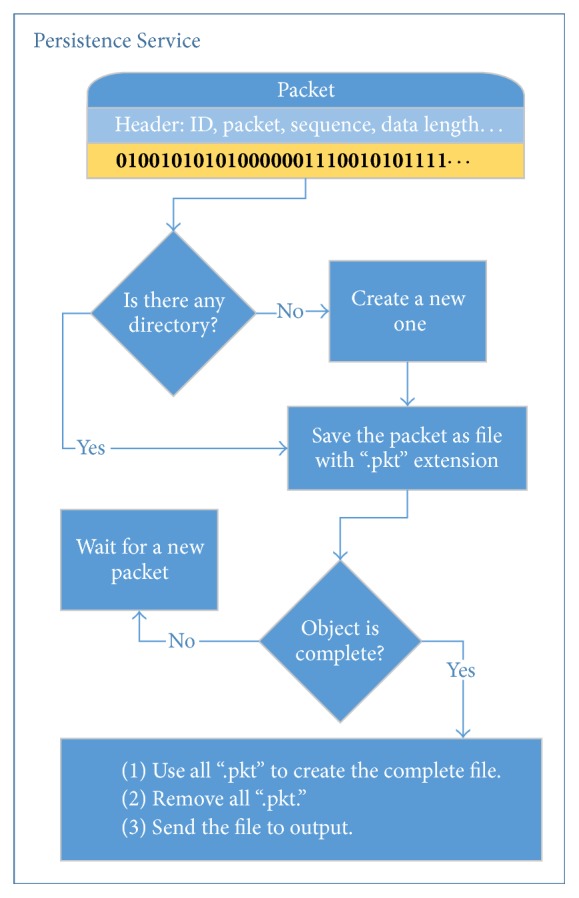
Persistence Service.

An output of the Consistency Checker is an input for the Persistence Service. This service aims to persist the data and notify stream events received from the Consistency Checker. For each received packet, the Persistence Service reads the header of the received packet. For this phase only the following fields are evaluated: ID, number of packets, packet sequence, and data length. To implement the Persistence Service some Java libraries were used for manipulating files. Among these libraries deserve mention File, FileWriter, BufferedWriter, BufferedReader, and FileReader. The entire process executed by the Persistence Service can be viewed in Figure 7.

The process of persistence starts by checking if there is a directory created to store the packets of an object. This is done through the ID field of the packet. If the directory does not already exist, a new one is created with the name of the object identifier. Then, in this directory, the data portion of the packet is persisted in a file whose name is the Packet Sequence field value and whose extension is “.pkt”. After that, the Persistence Service checks whether the object is complete (if the amount of files with the extension “.pkt” in the directory is equal to the Number of Packets field value). If the object is complete, files with the extension “.pkt” are read sequentially and their contents are persisted in a single file. At the end of this reading process, files with the extension “.pkt” are excluded and an object instance is sent to the output of the Persistence Service.

Whenever a stream event object is received, its descriptor is extracted and the Persistence Service takes the following steps:

1. It checks to tell that an event object with the equal event ID is present in the current object carousel. If an event with that event ID is not existent, then the descriptor is skipped.
2. If the data of the descriptor indicates that the event is a “do it now” event, subsequently the event is notified instantly;
3. If the event is not a “do it now” event, the Persistence Service verifies the NPT value at which the event should be notified. If an event with the same event ID is already scheduled to be notified at the same NPT value, or if the NPT value has already passed, then the event descriptor is skipped.
4. Once the NPT value reaches the value specified for a scheduled event, the event is notified.

One essential aspect that is offered by this division of stream event and stream event descriptors is that events can be reused. Some stream event descriptors can include the equal event ID, even if they are notified at different times and contain other private data. This lets an application programmer to use the event ID to define “classes” of events. Hence, an interactive TV application can start handling an event just by knowing the event ID. In several cases, no other data is needed to application

An output of the Persistence Service is used as input to the Receipt of Contents Notifier. It aims to inform the Presentation module that a particular object (interactive content) or stream event was received. This notification was implemented according to the observer design pattern [40] and use classes from “com.sun.dtv.broadcast.event” JavaDTV [10] package.

#### 4.2.2. Main Content Subscriber

This module aims to receive, encapsulate, and deliver the linear content to the presentation module. The implementation of this module used the same libvlc library included in the project Video LAN media player (VLC), which was presented in Section 4.1. The Main Content Subscriber was implemented into two submodules: Receiver VLCj and Container VLCj.

The Receiver VLCj aims to receive the linear content. For this, a user must define the communication protocol and the listening network ports. In the simulator, the protocol used for receiving audio/video streams is UDP and the listening port is an integer number that represents a TV channel.

Once the linear content is received, it serves as an input to the Container VLCj submodule. This submodule aims to integrate all the contents previously received in a container. After that, this container will be delivered to the Presentation module.

#### 4.2.3. Presentation

The Presentation module is responsible for providing an interface between the simulator and a user. In this module, the linear content and interactive content are presented together to the viewer. For this, the module was divided into two submodules: Main Content Presentation and Ginga-J Execution Engine.

The Execution Engine Ginga-J aims to present the JavaDTV applications. These applications are presented on a Java container (called Overlay) which is also used to present linear content. The remaining types of interactive content (videos, audio, images, text, etc.) will be consumed by these applications. The implementation of this module was based on the use and adaptation of the source code of the Ginga-J Emulator [9].

## 5. Experiments

This section presents the experiments conducted to demonstrate the BiS tool implemented in this work. These experiments use interactive applications to evaluate the tool with three important scenarios. They aim to demonstrate that the BiS tool supports the transmission of interactive content over a simulate broadcast channel. The three applications that have been developed will be detailed below.

### 5.1. Mixed Martial Arts

The first experiment consisted in developing and executing an interactive TV application, written in Java, which allows presenting interactive content coming from a broadcaster, during a transmission of MMA fights (Mixed Martial Arts). The application aims to present the photo of each fighter participating in a fight. These photos will be transmitted by a broadcaster and, once received, will be presented automatically. This experiment seeks to keep the focus on detailing the full operation of the simulator. These details include content selection, content transmission, content reception, and, finally, the presentation to a user.

**Figure 8:**
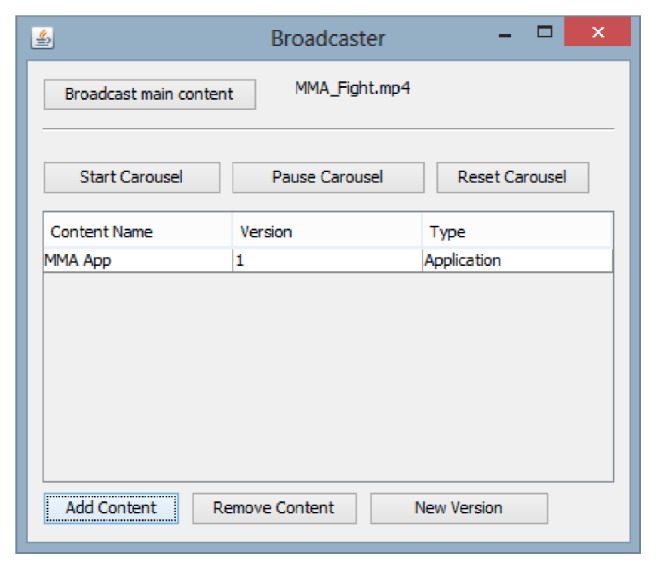
Broadcaster simulator window.

Before transmitting the stream that represents the linear content, the user must select a video file. This is done by using a button (named “Broadcast Main Content”) located on the top of the broadcaster simulator window. This window can be seen in Figure 8.

To transmit all the interactive contents (Java classes and photos of two fighters) through the data carousel, a user must first define the application name. In this experiment, this name was defined as MMA App (Figure 8). To send this content, the user must first click on the “Add Content” button and then select a file with extension “class” (the Java main class). After that, the simulator browses the directory where this file is stored and selects all other existing files (including subfolders) for transmission. Each file is mapped to a “Binded File” object and, moreover, the simulator also creates a “Binder Application” object. After that, the user must click on the “Start Carousel” button and then the transmission begins.

The “Binder Application” object created by the simulator aims to list all the objects that make up the MMA App application. The directory tree of the MMA APP application includes the Xlet “MMA.class” and the photos fighter1.png/fighter2.png that will be presented during transmission. Each of the objects generated by the simulator contains an identifier that is unique to that channel. Note that the “Binder Application” object contains all identifiers of objects that compose the application, the path to the Java main class (“MMA.class”), and an indication of how to treat each object (execute, use as data consumed by application, etc.).

**Figure 9:**
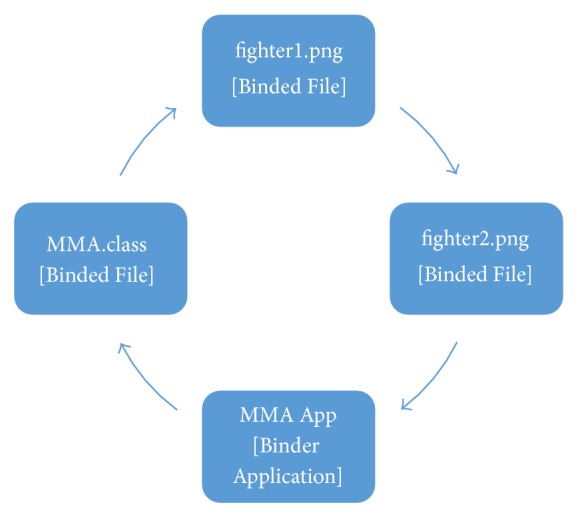
Cyclical sending of objects through the data carousel.

After the step described above, all objects are added to data carousel and sent cyclically using broadcast method.  Figure 9 represents the cyclical sending of objects through data carousel considering this experiment. For this, each object occupies a fixed time frame. When a time frame expires the next object is transmitted and this process is repeated successively until the last object of carousel. Once the last time frame has occurred, the carousel selects the first object again and repeats all the transmission cycles. The carousel stops sending objects when the user removes the application (thus removing all related objects) or when the user manually pauses or stops the carousel. The removal/replacement of objects can also be made during a transmission without stopping the carousel.

**Figure 10:**
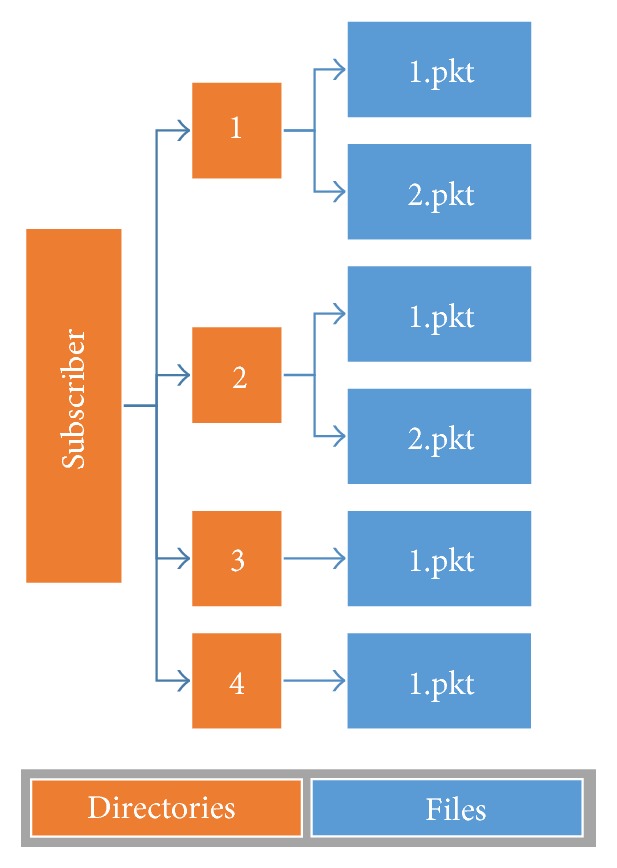
File structure of the simulator on receiver side.

The Java class files as well as the photos of the fighters are received in packets and stored in a temporary area on disk. For each object carousel, the simulator creates a folder named with its respective packet identifier. This folder stores the packets that are named with a sequence number and “.pkt” extension.  Figure 10 shows a file structure of the simulator (Receiver).

**Figure 11:**
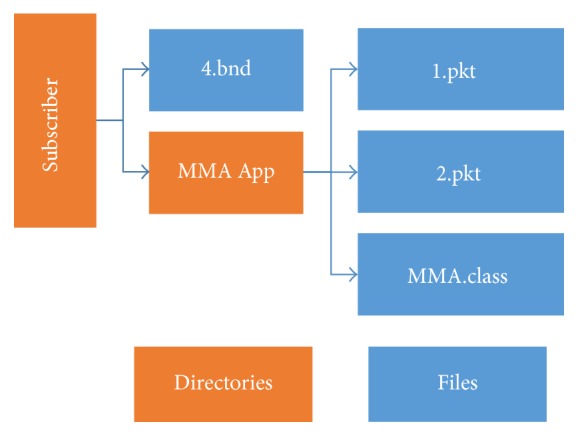
Structure of received files.

Each object received is treated in a differentiated way. “Binder Application” objects are stored in the root of the directory tree with naming formed by its ID and “.bnd” extension. For this experiment the object name is “4.bnd”. Then, the “Binder Application” object checks if there is any related file in the waiting list of “Binded Files” objects. These objects are stored on disk in a folder whose name is an attribute of the object itself. After that, two situations may occur: (1) if the “Binder Application” object has been received, it is informed through an event that the “Binded Files” objects associated with it have been received and can be consumed or (2) if the “Binder Application” object was not received the “Binded Files” objects are stored on a waiting list.  Figure 11 shows the directory tree for this experiment.

**Figure 12:**
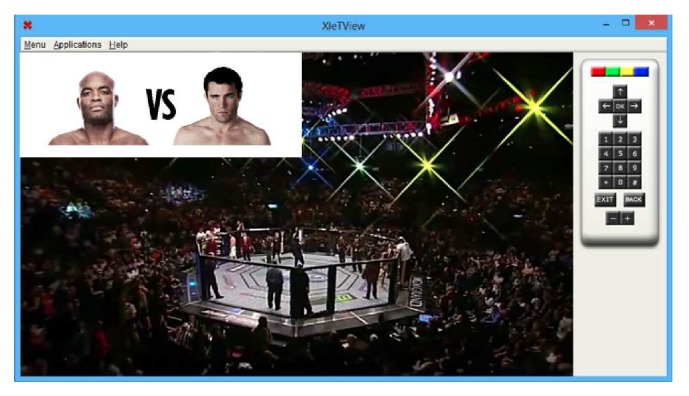
APP MMA execution on the simulator.

Once a “Binder Application” finds all objects linked to it in the list, or as soon as it receives all notification events, it triggers an event indicating that an application has just been received by the simulator. When the applications controller receives the triggered event, it will verify the type of execution of the application. In this experiment this type is* Automatic* (see Section 4.1.1).  Figure 12 shows the simulator during the broadcast of an MMA fight and the application that can present photos of the fighters.

### 5.2. Weather Forecast

Another experiment conducted in this work was the development of an interactive TV application in Java, named Weather Xlet, which allows a user to view a weather forecast during a transmission of a Digital TV show (e.g., a Formula 1 race). This application uses two different data sources (Broadcaster and Internet) and presents to the user information about the temperature at different locations. In this example, the temperature data in two Brazilian cities (Salvador and Rio de Janeiro) are fictitious. The experiment enables demonstrating the consumption of contents from a source other than broadcaster.

**Figure 13:**
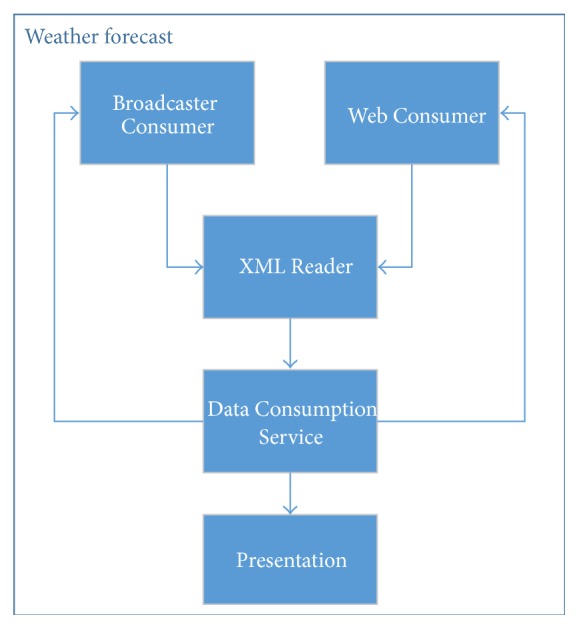
Weather Xlet architecture.

The architecture of this implementation (see Figure 13) was divided into five modules: Web Consumer, Broadcaster Consumer, XML Reader, Data Consumption Service, and Presentation.

The Web Consumer module performs HTTP Get requisitions and receives back an XML file whose content represents the temperature data of Salvador and Rio de Janeiro. The XML files received are sent to the XML Reader module. The development of this module used Java libraries for reading HTTP requests and data stream (e.g., HttpURLConnection, InputStream, and BufferedReader).

The Broadcaster Consumer module checks for a file (named clima.xml) in the received files folder (this file reaches this folder through the process explained in Section 4.2.1). If this file exists (already received) then it is sent to the XML Reader module.

The XML Reader module reads XML files sent by the Broadcaster Consumer or the Web Consumer and returns data (the name of each city and its respective temperature) to the Data Consumption Service module. The code below shows the structure of an XML file used in this experiment.

  <root>
   <climates>
    <climate>
     <local>Salvador</local>
     <temperature>29</temperature>
    </climate>
    <climate>
     <local>Rio de Janeiro</local>
     <temperature>40</temperature>
    </climate>
   </climates>
  </root>

The Data Consumption Service module is responsible for request data updates from both consumers. At the end of each data request, this module sends information to update the Presentation module. The implementation of this module uses an independent thread (other than the main thread) to avoid issues such as blocking its execution.

**Figure 14:**
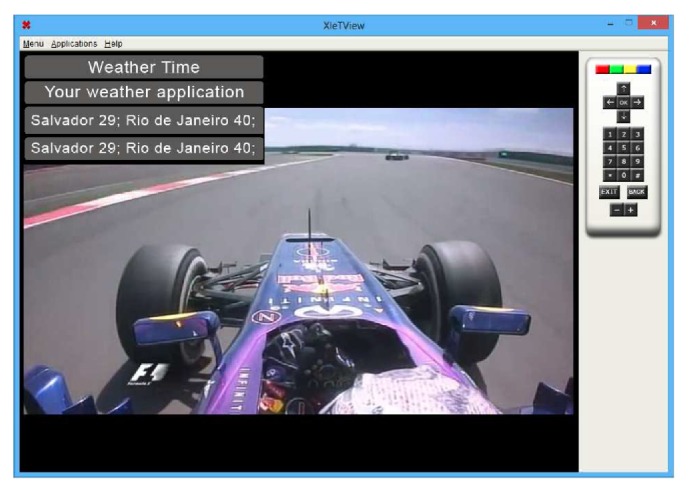
Weather forecast application that consumes data from the Broadcaster and the Internet (Weather Xlet).

The Presentation module receives data from the Data Consumption Service and presents it to a user using the LWUIT library (e.g., Form, Label). In Figure 14, the table rows shows (i) the title “Weather Time”; (ii) the message “Your weather application”; (iii) the forecast, sent by a broadcaster to Salvador (29 degrees) and Rio de Janeiro (40 degrees); and (iv) the same prediction obtained from the Internet content provider.

### 5.3. Video Replay

The last experiment of this study was, again, the development of an interactive TV application in Java, named Xlet Replay, which allows the user to view replays of videos while viewing a linear TV program. This application uses only a broadcaster as data source and presents to the user a menu that lets him select and present a snippet clipped from the main video.

In this example, the TV show and replays sent by the broadcaster are all about a F1 race. The occurrence of replays in F1 races is constant and is of high interest to the viewers. Another important aspect of this experiment is that it is not possible to know in advance the type of content of a replay during a F1 race. This situation occurs because in a live linear TV program it is not possible to determine when a highlight (a crash, a pit-stop, etc.) might happen.

The focus of the simulator is not the production phase. Thus, the videos with replays used in this experiment are already edited and stored on disk. In a real environment these videos could be created during the presentation of linear TV program and, of course, after the occurrence of the corresponding event (highlight). In this experiment, the synchronization constraints are built in presentation time. At every new Replay sent to the receiver, the restrictions need to be updated to allow the presentation of new interactive content.

**Figure 15:**
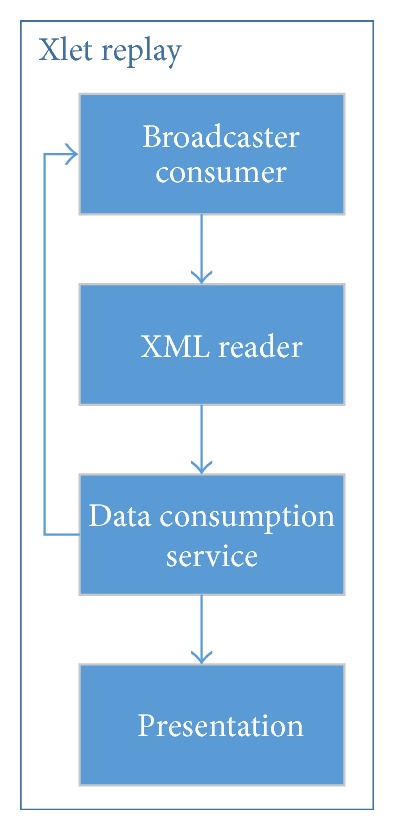
Xlet Replay architecture.

The architecture of this application (see Figure 15) is almost the same as the Weather Xlet (see Figure 13). Except for the Consumer Web, it has the other four modules: Broadcaster Consumer, XML Reader, Data Consumption Service, and Presentation. These four modules have the same role as before but in a different context. The first difference concerns the XML files contents. In this application, these files sent by the Broadcaster Consumer return the following data to the Data Consumption Service module:

1. a Replay sequence number;
2. a path to the video file;
3. a video title;
4. a short description of the video content.

The code below shows the structure of an XML file.

  <root>
   <replays>
    <replay>
     <title>Largada</title>
     <description>
      Largada do Grand Prix
     </description>
     <sequence>0</sequence>
    </replay>
   </replays>
  </root>

**Figure 16:**
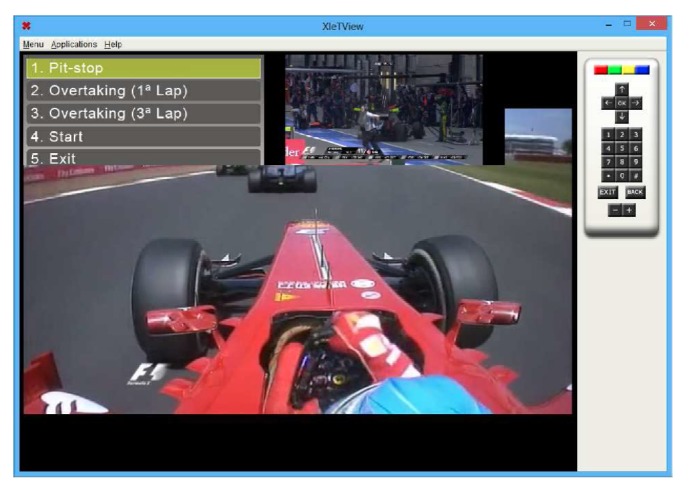
Xlet Replay main window.

After the XML Reader module has sent their response, the Data Consumption Service module sends the XML file content to update the Presentation module. This update means including new synchronization constraints on the presentation. The Presentation module receives these data and presents it to a user through the application window. This window presents a list with the name of each Replay so that a user can select and view. When a user selects a Replay, it will appear in a small area next to the list of replays available.  Figure 16 shows the execution window of this application.

## 6. Conclusion

This article presents a tool named BiS, Broadcast iDTV content simulator, for simulation of interactive TV applications. This simulator is distinguished from the other related tools on its wider scope because it allows simulating the transmission of applications along with the TV program (simulating the transmission of content and stream events over the air and in broadcast to the receivers), while the other tools perform only applications that are loaded locally from the repositories (e.g., USB and Hard Drive). Consequently, it is possible to incorporate several features using a communication protocol. This differential is a key point to make it possible to simulate the applications included in the categories defined in [2, 22, 28]. However, the BiS is comparable to existing proposals found in the literature since all of them support the execution of interactive applications. BiS has met all the requirements defined in Section 3.

Another aspect to be highlighted is that the BiS tool allows the execution of interactive applications in Digital TV, albeit not being limited to this platform. For example, interactive applications with focus on IPTV or Connected TV can use this environment since they also run over TCP/IP networks.

One of the difficulties in implementing this simulator was the construction of the communication mechanisms. To keep the similarity with the actual environment such mechanisms should be in accordance with the DSM-CC standard. However, unlike other communication protocols (e.g., TCP), we did not find (available for noncommercial use) an API that allows the use of DSM-CC for application deployment. This difficulty has become one of the contributions of this work: the definition and implementation of a communication protocol similar to DSM-CC (see Section 4.1.3).

One of the possibilities for further work includes the addition of support for the execution of other types of multimedia applications such as NCL, Flash, and SMIL. The BiS tool presented in this work includes only the execution environment for Java applications since its implementation is based on the execution engine Ginga-J. Another possibility for future work would be to adapt BiS to support multimedia applications witch support multiuser or multidevices. These applications allow the interaction of one (or more than one) user through mobile devices such as smartphones and tablets.

The presence of the test devices is very common in integrated development environments to make more efficient software. These devices may include, for example, simple text outputs (outputs), where the developer can monitor the outputs of the program or even include source code debugging devices. The simulation environment presented in this work has not the same purpose of an integrated development environment (applications coding). However, the integration of this simulator with some development environments like Eclipse or NetBeans as well the inclusion of such test devices could become another contribution of the BiS tool. The integration of these IDEs also opens the possibility of including the production activities as additional features of the simulator.

Another possibility for future work is to extend the simulator to allow the creation of multimedia applications with support for the transmission and reception of sensorial information in broadcast, as proposed by [41].

## Figures and Tables

**Table 1:** Tools versus phases involved in the creation, transmission, and presentation of iDTV content.

Tool/phase	Production	Transmission	Reception	Reproduction	Groups
Set-Top-Box Virtual Ginga-NCL	No	No	No	Yes	1 and 2
XleTView	No	No	No	Yes	1 and 2
OpenMHP	No	No	No	Yes	1 and 2
Ginga-J Emulator	No	No	No	Yes	1 and 2
FrameIDTV	No	No	No	No	—

**Table 2:** Protocol data packets header.

Field	Size
ID	8 bytes
Type	2 bytes
Version	2 bytes
Number of packets	8 bytes
Packet sequence	8 bytes
Data length	4 bytes
Data	30720 bytes

**Table 3:** Data field structure for Binder Application objects.

Bytes	Description
1	Type of execution (*Automatic*, *Notify*, *List*)
2	Size in *bytes* for the path to start class
3 to 252	Path to start class
253	Size in *bytes* for the application name
254 to 285	Application name
286 to 289	Number of objects
290 to 30720	Object IDs
